# Laparoscopic Isthmocele Repair: Efficacy and Benefits before and after Subsequent Cesarean Section

**DOI:** 10.3390/jcm10245785

**Published:** 2021-12-10

**Authors:** Stavros Karampelas, Georges Salem Wehbe, Laurent de Landsheere, Dominique A. Badr, Linda Tebache, Michelle Nisolle

**Affiliations:** 1 Department of Obstetrics and Gynecology, Centre Hospitalier Universitaire Brugmann, Université Libre de Bruxelles, 1020 Brussels, Belgium; Georges.SALEMWEHBE@chu-brugmann.be (G.S.W.); DominiqueBader@hotmail.com (D.A.B.); 2 Department of Obstetrics and Gynecology, CHR de La Citadelle, University of Liège, 4000 Liège, Belgium; ldelandsheere@chuliege.be (L.d.L.); linda.tebache@chuliege.be (L.T.); michelle.nisolle@chrcitadelle.be (M.N.)

**Keywords:** cesarean scar, uterine scar defect, laparoscopic isthmocele repair, niche, subsequent cesarean section

## Abstract

Objective: To evaluate the effect of laparoscopic isthmocele repair on isthmocele-related symptoms and/or fertility-related problems. The residual myometrial thickness before and after subsequent cesarean section was also evaluated. Design: Retrospective, case series. Setting: Public university hospital. Population: Women with isthmocele (residual myometrium < 5 mm) complaining of abnormal uterine bleeding, chronic pelvic pain or secondary infertility not otherwise specified. Methods: Women’s complaints and the residual myometrium were assessed pre-operatively and at three to six months post-operatively. In patients who conceived after surgery, the latter was measured at least six months after delivery by cesarean section. Main Outcome Measures: Resolution of the main symptom three to six months after surgery and persistence of laparoscopic repair benefits after subsequent cesarean section were considered as primary outcome measures. Results: Overall, 31 women underwent laparoscopic isthmocele repair. The success rates of the surgery as improvement of abnormal uterine bleeding, chronic pelvic pain and secondary infertility were 71.4% (10 of 14), 83.3% (10 of 12) and 83.3% (10 of 12), respectively. Mean residual myometrial thickness increased significantly from 1.77 mm pre-operatively to 6.67 mm, three to six months post-operatively. Mean myometrial thickness in patients who underwent subsequent cesarean section (N = 7) was 4.49 mm. In this sub-group, there was no significant difference between the mean myometrial thickness measured after the laparoscopic isthmocele repair and that measured after the subsequent cesarean section. None of these patients reported recurrence of their symptoms after delivery. Conclusion: Our findings suggest that the laparoscopic isthmocele excision and repair is an appropriate approach for the treatment of isthmocele-related symptoms when done by skilled laparoscopic surgeons. The benefit of this new surgical approach seems to persist even after a subsequent cesarean section. Further investigations and prospective studies are required to confirm this finding.

## 1. Background

Over recent decades, delivery by cesarean section (CS) has become increasingly common in both developed and developing countries. Nowadays, the percentage of CS delivery is well above the ideal rate recommended by the World Health Organization (WHO) [1,2]. In addition to short-term risks of CS, there are long-term risks several years after the current delivery that can affect women’s health and their future pregnancy outcomes [3].

The complications and consequences of CS are also increasing in number. Abnormal placentation and placenta previa have already been established and studied [4,5]. However, isthmocele, also termed cesarean scar defect or uterine niche, has recently been generating more interest [6,7,8,9,10,11,12].

Isthmocele is not a rare sequel of CS. Its exact incidence is unknown, but can reach 61% after primary CS and up to 100% following tertiary CS. In the majority of cases, it is asymptomatic and is diagnosed incidentally on transvaginal ultrasound scan (TVUS). In some cases, it can cause abnormal uterine bleeding, dysmenorrhea, dyspareunia, chronic pelvic pain or secondary infertility [2,12,13,14,15].

The management of isthmocele should be decided based on the patient’s symptoms and plans for future childbearing. It can be surgically treated using laparoscopy, hysteroscopy, vaginal repair or by combining hysteroscopy and laparoscopy. Studies and case reports have shown promising results in terms of success after surgical management, though the preferred surgical technique is still debated in the literature [16,17,18,19,20].

The laparoscopic repair of isthmocele consists in excising the fibrotic scar tissue followed by a two-layer closure with absorbable sutures. This management option can be considered for isthmocele with a residual myometrial (RM) thickness of <3 mm in women with a desire to conceive [17,18]. This was first described in 2003 by Jacobson et al. [20]. Nevertheless, there is a paucity of prospective studies analyzing the efficacy of this innovative technique in reducing patient’s symptoms and restoring women’s fertility. To date, the largest prospective study concerning the efficacy of the laparoscopic technique was conducted by Vervoort et al. in 2018 and included 101 patients. The authors concluded that laparoscopic repair was effective in reducing symptoms in most symptomatic women, but that more studies were needed to assess the real benefit of this procedure [18]. No published studies have shown whether the benefit of the scar repair persisted after a subsequent CS.

In the present article, we report our center’s experience and evaluate the effectiveness of laparoscopic isthmocele resection in a series of symptomatic women. The benefit of this intervention after a subsequent scheduled CS was also evaluated.

## 2. Materials and Methods

This was a single-center retrospective study (case series) performed between October 2016 and June 2020 in the Department of Obstetrics and Gynecology of the CHU, University of Liège, Belgium. The study was approved by the Hospital’s Ethics Committee under the reference number B412201836328. It included women with a history of at least one CS, who agreed to undergo a laparoscopic repair of their symptomatic isthmocele with a RM < 5 mm. In this series of patients, we also included patients treated laparoscopically for a cesarean scar ectopic pregnancy with a future desire to conceive.

Patients were considered symptomatic when they presented with one or more of the following entities: (1) abnormal uterine bleeding (AUB) defined as ≥2 days of intermenstrual bleeding or spotting, (2) chronic pelvic pain (CPP), which was defined as continuous or intermittent pain in the area below the umbilicus and between the hips and/or dyspareunia for at least six months and was not otherwise explained, (3) secondary infertility not otherwise specified.

Symptomatic women with the following conditions were excluded from the study: incomplete chart record, age < 18 years, a suspicion of genital tract malignancy, contraindications to general anesthesia, active cervical and/or pelvic infection, irregular menstrual cycles (cycle > 35 days or inter-cycle variation ≥ 2 weeks), diagnosis of cervical dysplasia, uterine polyps, cervical polyps, submucosal fibroids, diagnosis of endometrial atypia and intrauterine pregnancy.

Laparoscopic surgical treatment was not suggested to asymptomatic women with a large uterine scar defect and a desire to conceive. There is no evidence to suggest that surgical management of asymptomatic isthmocele improves obstetrical outcomes or reduces the risk of obstetrical complications.

### 2.1. Intervention, Procedures and Standard Care

All the included patients were informed of the risks and benefits of laparoscopic isthmocele excision and repair prior to surgery, of the potential need to convert to laparotomy during the operation, of the risk of intra-operative bladder injury, of bleeding and blood transfusion and of the possibility of post-operative infection and adhesion formation.

The patients were also asked to use adequate contraception post-operatively for at least 3 months to allow the uterine scar to heal properly. Women with a desire to conceive were advised to deliver by CS at 39 weeks during subsequent pregnancies since there is no strong evidence regarding the safety or the maternal and neonatal outcomes of the trial of labor or vaginal delivery after uterine scar defect excision and repair. A proper informed consent was signed prior to surgery by all women who met the inclusion criteria and accepted the proposed management.

One gynecologic surgeon performed the majority of the surgical procedures reported in this series (S.K., N = 23). The remaining operations were performed by two other surgeons (LT and LDL). All the surgeons had a surgical experience of more than five years in the field of minimal invasive gynecological surgery.

All the surgical interventions were done using the same surgical technique described in detail as follows [17,18]:

- The cesarean scar was easily identified by inserting a uterine probe through the cervix into the dehiscent scar. The vesicouterine peritoneum was detached and the bladder was safely separated from the uterus.
- Using a CO_2_ laser or cold scissors, the cesarean scar was completely opened from one side to the other. Excision of the fibrotic tissue from its edges was done, reaching healthy myometrium in order to enhance healing.
- In order to preserve the continuity of the cervical canal with the uterine cavity, a Hegar probe was inserted into the cervix before closing the uterine defect.
- Three separate X sutures using monofilament absorbable suture composed of poliglecaprone 25 (ETHICON/0-monocryl) were placed to close the deepest layer of the scar including the endometrium. A second superficial layer of running suture using a 2/0 monocryl was applied for a double-layer closure.
- In the presence of a retroverted uterus, a uterine anterior suspension procedure was performed by the retroperitoneal lateral suspension of the round ligament to the external oblique abdominal muscle aponeurosis. This was done in order to relieve the tension applied to the sutures, since the uterine retroversion may impair wound healing and predispose to the formation of scar defects.
- The same procedure was applied for ectopic scar pregnancies. The deficient uterine scar was excised en bloc with the adherent trophoblastic tissue.

### 2.2. Niche Measurements

The uterine and niche characteristics were assessed pre-operatively in all women using TVUS and/or saline infusion sonohysterography (SIS) and/or magnetic resonance imaging (MRI). Both MRI and ultrasound scan has been reported as of equal value for the detection and definition of uterine scar defects [12,17]. The post-operative uterine assessment was done using SIS, at one and 3 to 6 months after surgery.

In patients who conceived after laparoscopic isthmocele repair, the RM of the uterine scar was assessed six months after delivery by a scheduled CS using SIS. Niche depth and RM thickness were measured by certified sonographers on the sagittal plane where the niche was the largest (maximum depth, thinnest RM).

### 2.3. Description of the Subsequent Cesarean Section Surgical Procedure

All the surgical interventions were done using the same surgical technique. The hysterotomy was systematically done at the site of the previous uterine scar. The hysterorrhaphy was performed using a multifilament polyglactin suture with a double-layer closure technique. A profound and superficial layer of continuous unlocked sutures was performed.

### 2.4. Outcome Assessment

The primary outcome at 6-month follow up was defined as (1) complete resolution of AUB, (2) reduction or complete resolution CPP, and (3) spontaneous pregnancy.

### 2.5. Statistical Analysis

Statistical analysis was performed using SPSS 25 statistical software (IBM® SPSS® statistics, Endicott, New York NY, USA). Continuous variables were expressed as mean ± 1 standard deviation, while categorical variables were expressed as numbers (frequency), unless indicated otherwise. The Shapiro–Wilk test was used to check the normal distribution of continuous variables. We compared the means of related variables using either the t-test for related samples in the case of normal distribution or the Wilcoxon test when the distribution was not normal. Moreover, we compared the proportions of related variables using McNemar’s test. Thereafter, we performed a subgroup analysis of the patients who underwent cesarean delivery after their laparoscopic isthmocele repair using the same statistical methodology. However, we applied a post-hoc Bonferroni test to obtain the adjusted *p*-values. A *p*-value below 0.05 was considered statistically significant.

## 3. Results

From October 2016 to June 2020, a total of 38 symptomatic women with a large isthmocele were eligible and gave informed consent to undergo the proposed laparoscopic niche resection. We could define outcomes in 31 out of 38 patients. We excluded seven patients from the study (lost to follow-up).

Four of 31 patients (12.9%) had a cesarean scar ectopic pregnancy, and two of them reported CPP before conception. After removal of the pregnancy followed by the immediate repair of the defect, the patients reported resolution of their isthmocele-related symptoms.

A history of previous trial of labor of more than 5 h was noted in 17 patients out 31 (55%)

The baseline characteristics of the study population are summarized in Table 1.

In our series of 31 patients, isolated AUB was observed in 8/31 (25.8%), CPP in 6/31 (19.3%) and SI in 7/31 (22.5%).

Association of symptoms was observed in eight patients out of 31: 1/31 (3.2%) had AUB, CPP and SI; 2/31 (6.4%) had AUB and SI; 2/31 (6.4%) had CPP and SI; and 3/31 (9.6%) had AUB and CPP.

Following surgery, 10 out of 14 (71%) and 10 out of 12 (83%) patients had complete resolution of AUB and CPP, respectively, and spontaneous pregnancy was seen in 10 out of 12 (83.3%) participants presenting initially with SI.

**Figure 1:**
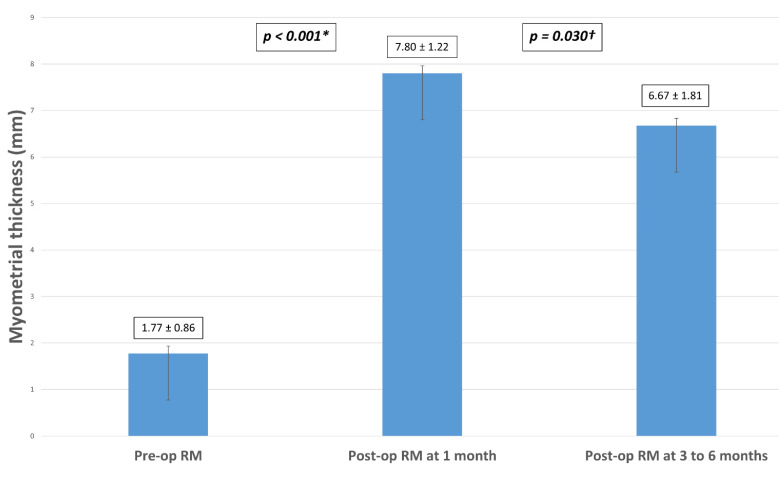
Measurements of mean RM thickness pre-operatively, at one month and 3 to 6 months after laparoscopic niche excision and repair in the study population (N = 31). Note: RM = residual myometrium; (*) = Significant difference between preoperative and postoperative residual myometrium measured at one month; (^†^) = Significant difference between postoperative residual myometrium at 1 month and 3–6 months.

In our study population, mean RM thickness increased significantly from 1.77 mm ± 0.86 pre-operatively to 7.80 mm ± 1.22 one month after the laparoscopic isthmocele excision and repair (*p*-value < 0.001). We noted a significant decrease in mean RM thickness from 7.80 mm ± 1.22 to 6.67 mm ±1.81 when it was measured three to six months after surgery (*p*-value = 0.03) (Figure 1). Nevertheless, it remained significantly higher than the pre-operative mean RM thickness (*p*-value < 0.001).

A sub-group analysis was done in patients who conceived spontaneously after laparoscopic isthmocele excision and repair and who underwent a subsequent CS at 39 weeks (N = 7). Mean RM thickness increased significantly from 1.73 mm ± 1.18 pre-operatively to 6.91 mm ± 2.66 at three to six months after isthmocele repair surgery (*p*-value < 0.001). The only adverse obstetrical outcome was a diagnosis of placenta previa in one patient. There was no uterine rupture or preterm labor in our series. None of these patients reported recurrence of isthmocele-related symptoms. RM thickness was assessed six months after delivery.

**Figure 2:**
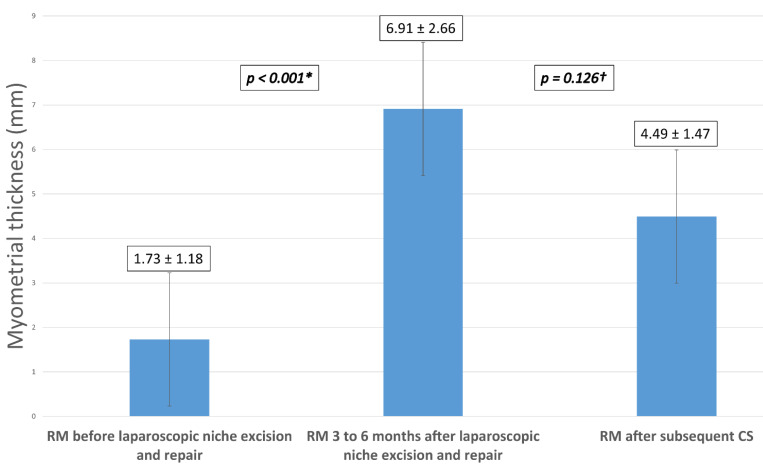
Sub-group analysis of mean RM thickness after subsequent CS in patients who conceived spontaneously after laparoscopic niche excision and repair (N = 7). Note: RM = residual myometrium, CS = cesarean section; (*) = Significant difference between the residual myometrium thickness before and 3 to 6 months after laparoscopic niche excision and repair; (^†^) = Nonsignificant difference between the residual myometrial thickness 3 to 6 months after laparoscopic niche repair and after the subsequent cesarean section.

Figure 2 is a histogram showing the sub-group analysis of mean RM thickness in these patients. There was no significant difference between mean RM thickness measured after the laparoscopic isthmocele repair (6.91 ± 2.66 mm) and that measured after the subsequent CS (4.49 ±1.47) (*p*-value = 0.126).

Table 2 lists the reported symptoms before and three to six months after laparoscopic isthmocele excision and repair and the success rates of the surgery as improvement in AUB, CPP and SI.

## 4. Discussion

There is a paucity of studies evaluating the effectiveness of laparoscopic niche resection for the treatment of symptomatic women with an RM thickness of <5 mm. Following the publications of Zhang et al., Donnez et al., and Vervoort et al., this study strengthens the evidence regarding the effectiveness of this relatively recent management strategy for symptoms associated with isthmocele [17,18,21].

In our study, we found high success rates of laparoscopic isthmocele excision and repair for the treatment of isthmocele-related symptoms (AUB, CPP and SI). Mean RM thickness increased significantly from 1.77 mm pre-operatively to 6.67 mm, three to six months post-operatively. Mean myometrial thickness in patients who underwent subsequent cesarean section was 4.49 mm. None of these patients reported recurrence of isthmocele-related symptoms after delivery. In this sub-group, there was no significant difference between the mean myometrial thickness measured after laparoscopic isthmocele repair and that measured after subsequent CS.

Our success rates regarding the treatment of AUB and CPP (71.4% and 83.3%, respectively) following laparoscopic isthmocele excision and repair are compatible with previously published studies [17,18]. A meta-analysis of the improvement of symptoms after laparoscopic/robotic treatment of isthmocele showed that symptom improvement ranged from 83.3% to 100%, with a very low rate of major iatrogenic complications [16].

The association between secondary infertility and isthmocele is well established. Cervical mucus, sperm quality and transport, and embryo implantation seem to be negatively affected by accumulation of intrauterine fluid and blood in the niche cavity. Fertility outcomes after uterine niche repair have been evaluated previously in several reports. Uterine scar defect repair is associated with high rates of fertility restoration. The results regarding the effect of laparoscopic resection of isthmocele are promising for the management of patients with fertility problems or for those undergoing fertility treatment. Vervoort et al. demonstrated objectively the disappearance of the accumulated intrauterine fluid and blood in most women after surgery. Moreover, the decreased niche depth and the increased RM might ease the correct insertion of catheters during embryo transfer [12,14,17,18,22,23,24].

In our study population, ten out of 12 patients with secondary infertility conceived spontaneously after surgery. These results again confirm the high rates of fertility restoration following laparoscopic isthmocele excision and repair.

In this case series, the significant increase in mean RM seen at three to six months after surgery suggests that laparoscopic isthmocele excision and repair is highly effective for adequate reinforcement of the myometrium. These findings are compatible with the findings of Donnez et al. and Vervoort et al. [17,18].

Upon histological analysis, the RM covering the niche was found to contain fibrotic tissue. The density of muscle fibers in this area was significantly lower than that of healthy myometrium adjacent to the scar. The resulting reduced contractility of the uterus impairs the drainage of menstrual blood and debris flow and predisposes women to AUB and SI. Consequently, adequate reinforcement of the abnormal myometrium at the niche level has a major impact on clinical outcome and symptom control [17,18].

The hysteroscopic correction of isthmocele may be the safest, easiest and fastest surgical treatment in patients with adequate residual myometrial thickness overlying the isthmocele (RM > 3 mm). The laparoscopic approach is the preferred option for patients with a thinner residual myometrium (RM < 3 mm) to avoid the risk of uterine perforation and bladder injury with the use of hysteroscopic resection of the defected uterine wall at the site of the niche. The mean value of the residual myometrial thickness in the study population is 1.77 mm. This was the main reason for which a laparoscopic approach was adopted. Moreover, for patients with a future desire of pregnancy, the laparoscopic surgical approach increases the residual myometrial thickness [16,17,18].

The significant decrease seen when comparing mean RM thickness at one month and three to six months post-operatively does not mean that there is a loss of therapeutic effect of surgery over time. RM thickness was still significantly higher than pre-operative mean RM thickness (*p*-value < 0.001). This finding can be related to resolution of the inflammatory changes and edema of the uterine myometrium at the surgical site that leads to overestimation when RM thickness is evaluated soon after surgery [25]. This observation suggests that it might be better to evaluate RM thickness at least three to six months after surgery in order to have an accurate assessment of the surgical therapeutic effect.

The benefit of laparoscopic repair of isthmocele seems to persist even after a subsequent CS. This is the first study to assess RM thickness in patients who conceived following laparoscopic isthmocele excision and repair and who delivered by a subsequent CS. These measurements were available only for seven patients. Nevertheless, this did not prevent us from identifying a certain trend in the outcomes since the preliminary results appeared to be logical and consistent with what has been established regarding the pathophysiology and risk factors of uterine scar defects [2,6,12,24,26].

History of multiple CS has been reported to be the principal risk factor linked to the development of isthmocele. Nevertheless, in our study, seventeen patients out of 31 (55%) had isthmocele following a primary CS. These CS were performed for different reasons, but in the majority of cases after a trial of labor. This finding highlights the involvement of other predisposing factors related to the obstetrical conditions at the time of CS, in addition to the surgical technique itself [2,6,12,21,24,25,26,27,28].

In addition to genetic predisposition, many other risk factors for developing uterine niche following CS have been proposed, the majority of which are surgically induced. The first is the theory of “low hysterotomy” or, in other words, when the uterine incision is made in the cervical tissue instead of the lower uterine segment. The mucus-producing glands present at this level may alter uterine wound healing. A dilated cervix is difficult to distinguish from the uterine wall. This may explain why a greater risk of isthmocele is found in patients undergoing CS with cervical dilation of more than 5 cm, longer duration of labor (>5 h) and lower fetal station [12]. Moreover, a systematic detachment of the vesicouterine flap may also lead to lower uterine incisions.

Incomplete closure of the uterine wall is another suggested risk factor. The double-layer hysterotomy closure using non-locking sutures is supposed to result in a thicker residual myometrium and a lower risk of a symptomatic isthmocele [2,6,12,21,24,25,26,27,28]. Nevertheless, Stegwee et al. found no difference in the number of days of postmenstrual spotting nine months after a first CS in women randomized to a single-layer versus double-layer unlocked uterine closure technique. Moreover, the single-layer technique was found to have some small benefits, such as shorter operative time, lower niche prevalence, fewer reported intercourse-related complaints, higher sexual satisfaction and less deterioration in general health and social functioning. In terms of societal costs, double-layer closure was not cost-effective [29].

It is evident that none of the previously cited risk factors, apart from genetic predisposition, are present in the case of a scheduled repeat CS following laparoscopic niche repair. In this particular context, the uterine cervix is not effaced or dilated, the patient is not in labor, and the fetus is not engaged. A higher incision of the uterine wall and double-layer closure of hysterotomy are performed during the scheduled CS. These factors may explain the persistence of the myometrial reinforcement and the lack of isthmocele symptoms after subsequent CS. A prospective analysis done on bigger patient population is needed to confirm this specific finding.

The surgical technique performed by our team was adopted from the reports published by Donnez et al., who were among the first to establish and to investigate this technique [17]. The use of a monofilament suture material was found to have a positive effect on uterine scar healing and on RM thickness when compared with a multifilament suture [28]. The same technique was applied for the en bloc laparoscopic excision of the deficient uterine scar and the adherent trophoblastic tissue in women diagnosed to have a cesarean scar ectopic pregnancy. Estimated intra-operative blood loss did not exceed 200–250 cc in any of the four patients. This volume does not significantly exceed the reported blood loss in ultrasound-guided trans-cervical suction curettage when the latter was adopted to manage cesarean scar ectopic pregnancy [30]. Moreover, the laparoscopic technique allowed the evacuation of the ectopic products of conception as well as the beneficial correction of the defect.

Various diagnostic modalities such as TVUS, SIS, hysterography, hysteroscopy and MRI are used for the diagnosis of isthmocele. Nevertheless, the assessment of RM can be made only by using TVUS, SIS, and MRI. All the patients included in our study underwent pre-operative evaluation of RM using TVUS, SIS and/or MRI. Since SIS is more affordable than MRI and has a greater sensitivity and specificity compared to TVUS for the identification of isthmocele and the assessment of RM, it was adopted in our study protocol for the post-operative follow-up [12,31,32,33,34,35,36].

The most reasonable hypothesis regarding the pathogenesis of cesarean scar ectopic pregnancy is that the conceptus enters the myometrium through a dehiscent tract or a defect. Significant association between cesarean scar ectopic pregnancy and uterine scar defect has been reported. Cesarean scar defect was seen in up to 70% by hysteroscopy, after hysteroscopic removal of ectopic tissue of conception [37]. These findings suggest that scar ectopic pregnancy is a strong indicator of a pre-existing symptomatic or asymptomatic cesarean scar defect. Two out of four patients with ectopic pregnancies involved in our study (50%) had chronic pelvic pain which is a well-known clinical presentation of isthmocele. This observation supports the idea of the “pre-existing” uterine scar defect pre-disposing to cesarean scar ectopic pregnancies.

The assessment of the pre-operative myometrial thickness could be potentially altered by the pressure effect of the ectopic gestational sac growing within the uterine scar. Nevertheless, cesarean scar pregnancy is a rare form of ectopic pregnancy and there is a paucity of scientific literature that can provide us with clear and strong evidence concerning the impact of gestational sacs on the accuracy of the assessment of the pre-operative myometrial thickness.

In our tertiary referral university hospital, the majority of cesarean scar ectopic pregnancies are treated surgically, especially in patients with a desire of future pregnancy. We use laparoscopy to excise the deficient uterine scar “en bloc” with the adherent trophoblastic tissue. This surgical approach is useful not only to remove the ectopic tissue of conception, but also to correct the pre-existing uterine scar defect and to increase the residual myometrial thickness within the same procedure.

The main limitation of our study was the small number of patients who were eligible for inclusion in the analysis. The final population of women that could be followed up after a subsequent CS was small (N = 7). Nevertheless, we could identify a trend in the outcomes and preliminary results that appeared to be logical and clearly consistent with what has been described in the literature. One more limitation of this study is that the pre-operative assessment was not homogeneous. A single pre-operative diagnostic protocol will be adopted in future prospective studies. Another drawback is that the time of follow-up in this study was limited, and it would be interesting to gain more long-term data on the studied population.

## 5. Conclusions and Implications for Practice

Our study findings suggest that laparoscopic isthmocele excision and repair seem to be an appropriate approach for the treatment of isthmocele-related symptoms when done by skilled laparoscopic surgeons. The benefit of this new surgical approach seems to persist even after a subsequent CS. Further investigations and prospective studies are required to confirm this finding and to define the long-term gynecological and obstetrical outcomes of this procedure.

## Figures and Tables

**Table 1:** Baseline characteristics of the study population.

Patient	Age	Previous Trial of Labor of > 5 h	Previous CS	Symptoms	Pre-op RM (mm)	Post-op RM at 1 Month (mm)	Post-op RM at 3–6 Months (mm)	Spontaneous Pregnancy after Laparoscopic Repair	RM after Subsequent CS (mm)
1	31	+	3	AUB	1.5	6	8.2	+	NA
2	48	+	2	AUB	2.2	6.5	NA	−	−
3	24	+	1	SI	0.6	NA	9.2	+	3.6
4	33	+	1	SI	1.4	8.5	8	+	5.2
5	32	−	1	SI	2.3	8.5	7.8	+	4
6	37	−	3	AUB	1.5	7.5	5.6	−	−
7	30	+	2	AUB	1.7	5.8	3.7	−	−
8	38	+	1	CPP	1.9	8.2	7.4	−	−
9	44	−	1	AUB	2.1	8.7	7.2	−	−
10	47	−	1	AUB	1.3	7	6,4	−	−
11	41	−	4	AUB-CPP	2.2	8.6	NA	−	−
12	25	+	1	SI	1.3	9.1	9.6	+	6.8
13	24	+	1	SI	2.9	7.6	4.5	+	NA
14	38	+	2	AUB-CPP-SI	1.9	8.4	7	+	5
15	33	+	1	AUB	2.3	NA	7	+	NA
16	32	+	1	SI	1.4	5.3	5.3	+	NA
17	39	−	1	AUB-SI	4.7	7	NA	+	NA
18	36	−	1	AUB-SI	4	NA	4.7	+	4.7
19	35	−	4	SI	1.9	NA	8.1	−	−
20	35	−	3	CPP-SI	0.6	NA	2.1	+	2.1
21	37	+	1	AUB	1.5	9.3	8.5	+	NA
22	29	+	1	CPP	1.5	10	7	+	NA
23	44	+	2	AUB-CPP	1	9.2	NA	−	−
24	32	+	1	AUB-CPP	1.8	NA	NA	−	−
25	45	+	3	CPP	1.9	8	6	−	−
26	29	−	1	CPP-SI	1.4	8	8	−	−
27	32	−	1	CPP	1.8	6.4	NA	−	−
28	34	−	4	CPP-CS scar ectopic pregnancy	1	NA	NA	−	−
29	42	−	3	CPP-CS scar ectopic pregnancy	1	7.7	6.4	−	−
30	40	+	2	CS scar ectopic pregnancy	1.4	NA	5.6	−	−
31	36	−	2	CS scar ectopic pregnancy	1	8.2	NA	−	−

Note: AUB = abnormal uterine bleeding; CPP = chronic pelvic pain; SI = secondary infertility; CS = cesarean section; RM = residual myometrium; NA = not available. mm = millimeter; (+) = YES; (−) = NO.

**Table 2:** Percentages of the reported patient’s symptoms before and 3 to 6 months after laparoscopic isthmocele excision and repair and success rates of the surgery as improvement in AUB, CPP and SI.

	Before Laparoscopic Isthmocele Excision and Repair N = 31	3 to 6 Months after Laparoscopicisthmocele Excision and Repair N = 31	Success Rate	*p*-Value
**AUB**	14 (45.2%)	4 (12.9%)	71.4%	0.002
**SI**	12 (38.7%)	2 (6.5%)	83.3%	0.002
**CCP**	12 (38.7%)	2 (6.5%)	83.3%	0.002

Note: AUB = abnormal uterine bleeding; CPP = chronic pelvic pain; SI = secondary infertility.

## Data Availability

The data presented in this study are available on request from the corresponding author.
